# Adjuvant Mood Stabilisers for Long-Term Management of Treatment-Resistant Schizophrenia: A Case Report

**DOI:** 10.7759/cureus.104153

**Published:** 2026-02-23

**Authors:** Fumnanya Anyameluhor, Chloe Elliott

**Affiliations:** 1 Psychiatry, Elysium Healthcare, Mansfield, GBR; 2 Psychology, Elysium Healthcare, Mansfield, GBR

**Keywords:** case report, case series, clozapine therapy, difficult-to treat schizophrenia, inpatient psychiatric rehabilitation, lamotrigine augmentation, multidisciplinary care, pharmacological optimization, treatment-resistant schizophrenia, valproate augmentation

## Abstract

Treatment-resistant schizophrenia (TRS) is associated with persistent psychotic symptoms, protracted hospital admissions, and significant functional impairment despite adequate trials of antipsychotic treatment. Optimisation of antipsychotic medication, particularly clozapine-based treatment regimens, alongside multidisciplinary inpatient rehabilitation, is one of the mainstays for improving outcomes in difficult-to-treat schizophrenic presentations. Persistent symptoms despite clozapine treatment have been shown to improve with adjunctive mood stabilisers, especially when there is affective instability.

This retrospective case report describes individuals with long-standing TRS receiving care in an inpatient rehabilitation service. Adjunctive mood stabiliser medication was introduced as part of pharmacological optimisation alongside structured rehabilitative interventions for maximum symptom control. Following this approach, gradual improvements were observed in symptom control and engagement with rehabilitation. These cases highlight the potential benefit of mood stabiliser augmentation, such as Lamotrigine or Valproate, in difficult-to-treat presentations, particularly where there is a prominent affective component. Integrated multidisciplinary inpatient rehabilitation and pharmacological optimisation are essential for achieving sustained symptom control and functional recovery in complex psychiatric presentations.

## Introduction

Treatment-resistant schizophrenia (TRS) has been noted to affect about a third of individuals with schizophrenia [1-3] and is often associated with increased morbidity and generally poor functional outcomes. Clozapine remains the gold standard pharmacological treatment option [4]; however, residual symptoms and, at times, adherence challenges persist [5]. Augmenting antipsychotic medications with mood stabilisers such as Lamotrigine, particularly when there is an affective element, has been trialled in partial responders [6]. These strategies, alongside multidisciplinary interventions, are aimed at improving functional recovery as well as ameliorating long-term outcomes.

We discuss three service users with long-standing TRS managed in a male inpatient rehabilitation unit. This article highlights pharmacological interventions, the impact of physical health on treatment modalities, and rehabilitation outcomes.

Methods

This retrospective case report examined three service users with TRS who were receiving long-term care in an inpatient rehabilitation service. Data were extracted from multidisciplinary clinical records, including psychiatric reviews, nursing documentation, physical health monitoring, occupational therapy reports, and psychology input. Service users were anonymised to preserve confidentiality.

## Case presentation

Service User X

Service User X is a middle-aged male with a long-standing diagnosis of treatment-resistant paranoid schizophrenia, detained under a forensic section of the Mental Health Act since the mid-1990s. His illness followed a chronic, relapsing, and remitting course characterised by persistent auditory hallucinations, paranoid and grandiose delusions, and acute behavioural disturbance during periods of relapse. Recurrent exacerbations of psychotic symptoms, progressive functional decline over time, and associated risks necessitated ongoing inpatient treatment. Despite multiple adequate trials of antipsychotic medications, including haloperidol (discontinued due to an adverse reaction), olanzapine (20 mg/day), and risperidone (5 mg/day), symptoms persisted, and sustained remission was not achieved (Table 1). He was subsequently commenced on clozapine due to treatment resistance. Although some symptomatic improvement was observed, clozapine was discontinued because of poor tolerability. In addition to treatment resistance, his management was further complicated by physical comorbidities, including recurrent hyponatraemia, gait instability, obesity, and diabetes mellitus. As antipsychotic treatment alone did not adequately control impulsivity, affective instability, and aggression, sodium valproate was introduced as an adjunctive mood stabiliser at a dose of 800 mg twice daily, alongside ongoing antipsychotic treatment with risperidone (5 mg/day) and sulpiride (up to 1600 mg/day). Plasma levels were monitored as part of routine therapeutic optimisation. Following pharmacological optimisation and structured inpatient rehabilitation, there was modest behavioural stabilisation maintained over approximately six years of follow-up, with improved control of psychotic symptoms. There was also increased engagement in rehabilitative activities offered on the unit.

**Table 1:** Medication Timeline for Service User X

Medication sequence	Clinical response
Haloperidol/Olanzapine/Risperidone	No sustained remission
Clozapine	Partial improvement; discontinued due to intolerance
Sulpiride commenced + Risperidone restarted	This was due to clozapine intolerance and led to some improvement in psychotic symptoms
Adjunctive sodium valproate	Reduced aggression and behavioural disturbance

Service User Y

Service User Y is a young adult male with a diagnosis of schizophrenia and comorbid autism spectrum disorder, detained under a civil section of the Mental Health Act. The onset of illness occurred in late adolescence and was complicated by polysubstance misuse, predominantly cannabis and alcohol, with historical use of cocaine and MDMA (3,4-methylenedioxymethamphetamine), alongside limited insight into the impact of substance misuse on his mental health. His schizophrenia was characterised by long-standing persecutory and bizarre delusions, auditory hallucinations, formal thought disorder, and paranoia. He showed a poor response to two adequate trials of antipsychotic medication, namely risperidone 6 mg/day and olanzapine 20 mg/day, consistent with treatment-resistant illness, prompting the initiation of clozapine. There was substantial improvement when clozapine was commenced and titrated to 350 mg/day; however, this response was short-lived due to non-adherence. A subsequent re-trial of clozapine initially showed some improvement but plateaued after several months (Table 2). His clinical presentation was further complicated by behavioural dysregulation, which could not be accounted for by psychosis alone; consequently, lamotrigine was introduced as an adjunctive mood stabiliser to address his emotional volatility. Lamotrigine was gradually titrated to 100 mg/day. Following lamotrigine augmentation, there was a gradual reduction in affective reactivity and behavioural instability, alongside improved engagement with structured ward activities, while residual psychotic symptoms persisted at a lower intensity. At approximately six months following initiation of lamotrigine, although Service User Y continued to experience residual grandiose delusional beliefs, there was improved stability, better self-care, and increased participation in rehabilitative activities. 

**Table 2:** Medication Timeline for Service User Y

Medication	Clinical response
Risperidone/Olanzapine	Poor response
Clozapine (initial)	Marked improvement; relapsed due to non-adherence
Clozapine (re-trial)	Partial response; then plateaued
Lamotrigine (adjunctive)	Improved affective stability and functioning

Service User Z

Service User Z is an older adult male detained under a civil section of the Mental Health Act, with a long-standing history of schizophrenia complicated by recurrent severe depressive episodes and marked suicidality. His schizophrenia was characterised by persistent auditory hallucinations, long-standing delusional beliefs, and behavioural disturbance. This often fluctuated, with periods where his psychotic symptoms and aggression alternated with phases of withdrawal and nihilistic ideation. He previously achieved a prolonged period of clinical stability over several years while treated with Clozapine 400 mg/day in combination with lithium. Lithium was prescribed for a prolonged period and titrated to maintain therapeutic serum levels consistent with a moderate-to-high maintenance dosing range (Table 3). However, both were discontinued due to adverse effects, including lithium-associated renal impairment progressing to stage 4 chronic kidney disease, which required hospitalisation. He also had a period of life-threatening physical deterioration due to recurrent food and fluid refusal. His physical health co-morbidities limited medication options. A trial of depot and oral risperidone, up to 6 mg/day oral equivalent, was explored but did not alleviate symptoms; hence, a mood stabiliser was considered due to affective instability. Lithium was deemed unsuitable due to prior intolerance; hence, lamotrigine was initiated and gradually titrated to 200 mg/day as an adjunctive treatment. Following lamotrigine augmentation, and over approximately 12 months of follow-up, there was a reduction in the severity of behavioural disturbance, alongside a reduction in depressive symptom intensity, particularly nihilistic ideation and affective lability. In terms of schizophrenic symptoms, while core psychotic features such as delusional beliefs and hallucinations persisted, their intensity and associated distress were reduced, with fewer episodes of aggression. In combination with structured inpatient rehabilitation, these changes gradually led to improved engagement in psychosocial and therapeutic activities offered on the unit (Table 4). 

**Table 3:** Medication Timeline for Service User Z

Medication	Clinical response
Clozapine + lithium	Prolonged stability; discontinued due to adverse effects
Oral and depot risperidone	Limited symptom control
Lamotrigine (adjunctive)	Reduced depressive severity and improved engagement

**Table 4:** Summary Table Comparing the Medication History and Clinical Outcomes of the Three Service Users MHA: Mental Health Act.

Characteristic	Service User X	Service User Y	Service User Z
Age group	Middle-aged adult	Young adult	Older adult
Primary diagnosis	Treatment-resistant paranoid schizophrenia	Treatment-resistant schizophrenia with autism spectrum disorder	Treatment-resistant schizophrenia with recurrent depressive episodes
Legal status	Forensic section of the MHA	Civil Section of MHA	Civil Section of MHA
Duration of illness	>30 years	~10 years	>35 years
Antipsychotics trialled	Haloperidol, olanzapine, risperidone, clozapine	Risperidone, olanzapine, clozapine	Clozapine, lithium, risperidone
Current antipsychotic regimen	Risperidone and sulpiride	Clozapine	Oral and depot risperidone
Adjunctive mood stabiliser	Sodium valproate	Lamotrigine	Lamotrigine
Mood stabiliser dosing/monitoring	Titrated to therapeutic range; plasma levels monitored	Titrated per standard protocol; clinical monitoring	Titrated per standard protocol; clinical monitoring
Affective features	Impulsivity, irritability, aggression	Emotional volatility, behavioural dysregulation	Severe depressive symptoms, suicidality
Clinical change after augmentation	Reduced aggression and behavioural disturbance	Improved affect regulation and behavioural stability	Reduced depressive severity and behavioural disturbance
Follow-up after augmentation	Several years	Approximately 12 months	>12 months
Functional outcome	Improved engagement in rehabilitation	Improved self-care and social engagement	Improved engagement in psychosocial rehabilitation

## Discussion

These cases highlight the complex interplay between treatment resistance of psychiatric symptoms, physical health comorbidities, and rehabilitation in inpatient settings.

Clozapine remained central to treatment, demonstrating its superiority in TRS [4]. Despite this, relapse following clozapine discontinuation highlights the vulnerability associated with non-adherence and treatment intolerance. A re-trial with adjunctive lamotrigine illustrates the potential utility of augmentation strategies, alongside the importance of sustained engagement and adherence-focused strategies, as evident in Service User Y.

For Service Users X and Z, despite the superiority of clozapine in treatment-resistant illness and its initial improvement of symptoms, treatment could not be continued due to adverse reactions and medical comorbidities. Augmentation with mood stabilisers alongside second-generation antipsychotics was associated with improvements in affective stability, behavioural regulation, and functional engagement in rehabilitative therapeutic activities.

Adjunctive mood stabiliser use across all three service users was associated with affective stabilisation and overall improvement in mental state. Randomised controlled trials [6] suggest that lamotrigine augmentation of clozapine may lead to modest improvements in symptom severity in clozapine-resistant schizophrenia, particularly with respect to positive and affective symptoms. This supports lamotrigine as a promising pharmacological augmentation option in TRS and suggests its consideration in individuals for whom clozapine monotherapy is insufficient or poorly tolerated.

Physical health comorbidities also significantly influence treatment options and psychiatric stability, emphasising the need for integrated care. Multidisciplinary rehabilitative and pharmacological interventions facilitated gradual functional recovery in all three cases, despite residual psychotic symptoms. The non-linear clinical trajectories across the three cases reflect the enduring and heterogeneous nature of TRS, necessitating flexible, individualised treatment approaches.

Overall, these findings reflect the effectiveness of combined rehabilitative and pharmacological interventions in complex treatment-resistant presentations.

## Conclusions

TRS requires multidisciplinary management to increase functional outcomes. Despite Clozapine being the mainstay of its treatment, adjunctive mood stabilisers may offer additional benefits in some groups of service users, particularly those with prominent affective instability. The role of pharmacological and integrated multidisciplinary interventions is essential for promoting functional outcomes and reducing risks in the difficult-to-treat schizophrenic population.
